# QRS detection and classification in Holter ECG data in one inference step

**DOI:** 10.1038/s41598-022-16517-4

**Published:** 2022-07-25

**Authors:** Adam Ivora, Ivo Viscor, Petr Nejedly, Radovan Smisek, Zuzana Koscova, Veronika Bulkova, Josef Halamek, Pavel Jurak, Filip Plesinger

**Affiliations:** 1 Institute of Scientific Instruments of the Czech Academy of Sciences, Brno, Czech Republic; 2 Medical Data Transfer, s.r.o., Brno, Czech Republic

**Keywords:** Computational models, Cardiology, Computational science

## Abstract

While various QRS detection and classification methods were developed in the past, the Holter ECG data acquired during daily activities by wearable devices represent new challenges such as increased noise and artefacts due to patient movements. Here, we present a deep-learning model to detect and classify QRS complexes in single-lead Holter ECG. We introduce a novel approach, delivering QRS detection and classification in one inference step. We used a private dataset (12,111 Holter ECG recordings, length of 30 s) for training, validation, and testing the method. Twelve public databases were used to further test method performance. We built a software tool to rapidly annotate QRS complexes in a private dataset, and we annotated 619,681 QRS complexes. The standardised and down-sampled ECG signal forms a 30-s long input for the deep-learning model. The model consists of five ResNet blocks and a gated recurrent unit layer. The model's output is a 30-s long 4-channel probability vector (no-QRS, normal QRS, premature ventricular contraction, premature atrial contraction). Output probabilities are post-processed to receive predicted QRS annotation marks. For the QRS detection task, the proposed method achieved the F1 score of 0.99 on the private test set. An overall mean F1 cross-database score through twelve external public databases was 0.96 ± 0.06. In terms of QRS classification, the presented method showed micro and macro F1 scores of 0.96 and 0.74 on the private test set, respectively. Cross-database results using four external public datasets showed micro and macro F1 scores of 0.95 ± 0.03 and 0.73 ± 0.06, respectively. Presented results showed that QRS detection and classification could be reliably computed in one inference step. The cross-database tests showed higher overall QRS detection performance than any of compared methods.

## Introduction

**Figure 1:**
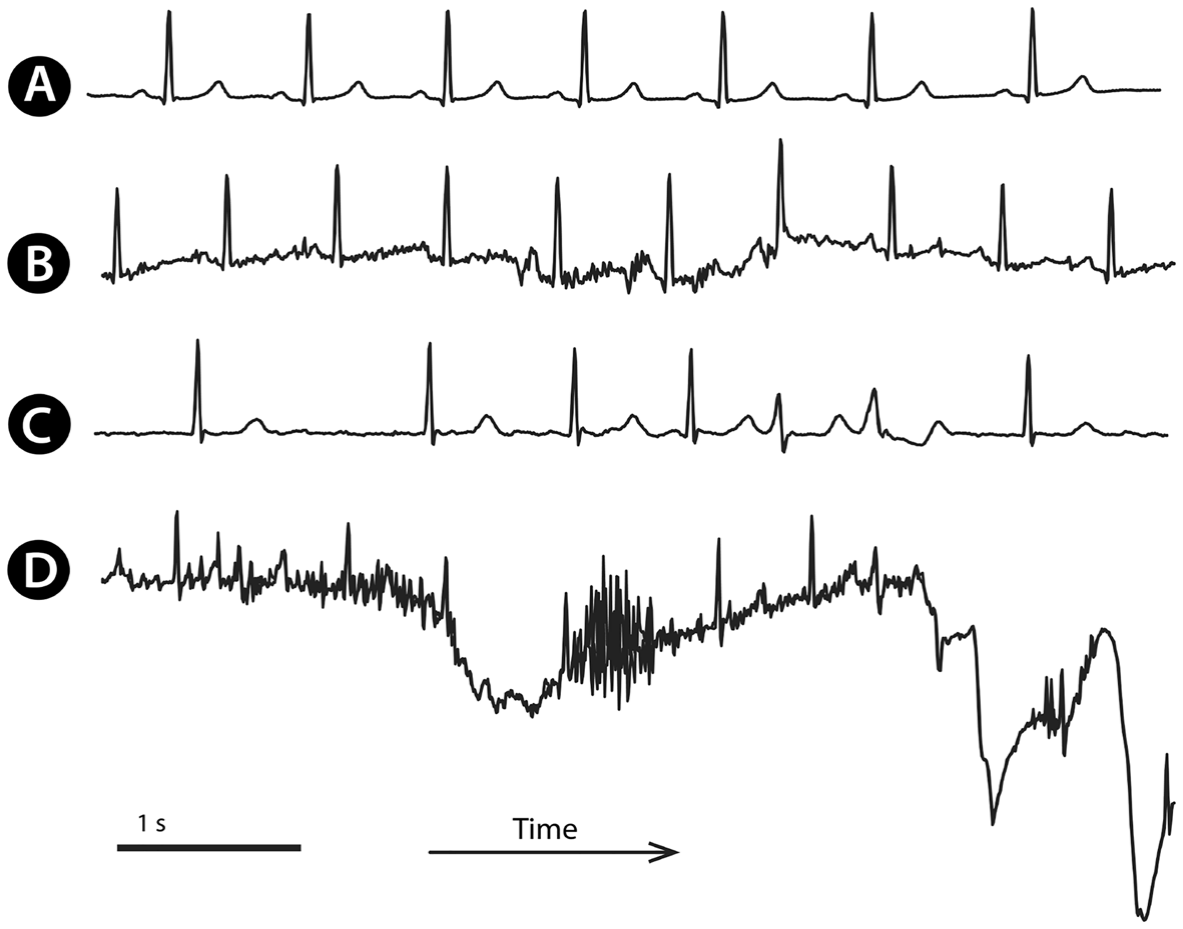
Examples of Holter ECG signals: Normal sinus rhythm (**A**); the same patient with the same rhythm, but disturbed by noise due to patient movements (**B**). A different patient with atrial fibrillation and premature ventricular contractions (PVC) in a couplet (**C**); the same patient a few minutes later, with disturbed ECG signal (**D**) with at least one PVC.

The electrocardiograph (ECG) is a common method to analyse heart rhythm and its disturbances. While some arrhythmias (such as atrial fibrillation in Fig. 1C,D) may be episodical, a patient can be equipped with a Holter ECG device to record longer periods (from 24 h to several days or weeks). Alternatively, a patient may be equipped with a wearable device to check the rhythm permanently. In both scenarios, the entry point in clinical analysis of Holter ECG data is reliable beat (i.e., QRS complex) detection and classification (Fig. 1) to describe patient rhythm.

### QRS detection

Existing beat detection methods are based on morphology analysis as QRS slope, amplitude, and width^1^, digital filtering^2–6^, wavelet transform^7^, machine learning^8,9^ or deep-learning^10,11^ approaches. These QRS detection methods performed great on public datasets as an MIT-BIH^12^. However, the Holter ECG data acquired during daily activities might still be challenging. The data contains a higher amount of noise caused by patient movements (Fig. 1B,D), further affected by the quality of electrode placement since during long-term Holter monitoring, subjects often place electrodes by themselves.

### QRS classification

**Figure 2:**
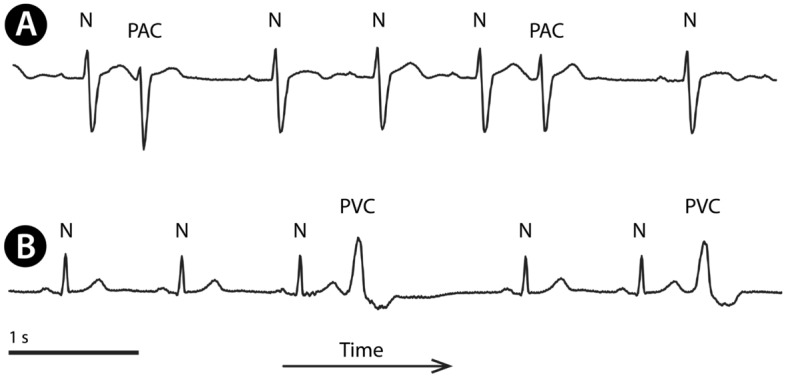
Examples of ECG signal with the most common QRS types: normal sinus beats (N), premature atrial contractions (**A**, PAC), and premature ventricular contractions (**B**, PVC).

Knowing QRS positions allows for evaluating heart-rate variability, minima, maxima, or the presence of pauses. If more precise ECG analysis is required, the most common beat classes can be recognised (Fig. 2): normal beats, premature atrial contractions (PAC), or premature ventricular contractions (PVC). When these beat classes are known, pathologies formed by specific beat sequences can be analysed. Then, for example, we can automatically identify PVC couplets, triplets, sustained or persistent ventricular tachycardia, or runs of supraventricular tachycardia.

Existing classification approaches may be based on engineered features^13,14^ or, nowadays, on deep learning techniques^15,16^, further implementing convolutional^17,18^ or recurrent layers^19,20^. Most of them are trained and tested using the public MIT-BIH database (47 patients) or CPSC database (2000 patients), both recorded in resting supine position. These methods are supposed to work using preceding QRS detection. However, deep-learning techniques allow the preparation of models covering QRS detection and classification in one inference step. Thus, we present a robust, deep-learning method to detect and classify QRS complexes in ECG data recorded during usual daily activities. We also introduce a novel approach, delivering detection and classification results in one inference step.

## Data

**Figure 3:**
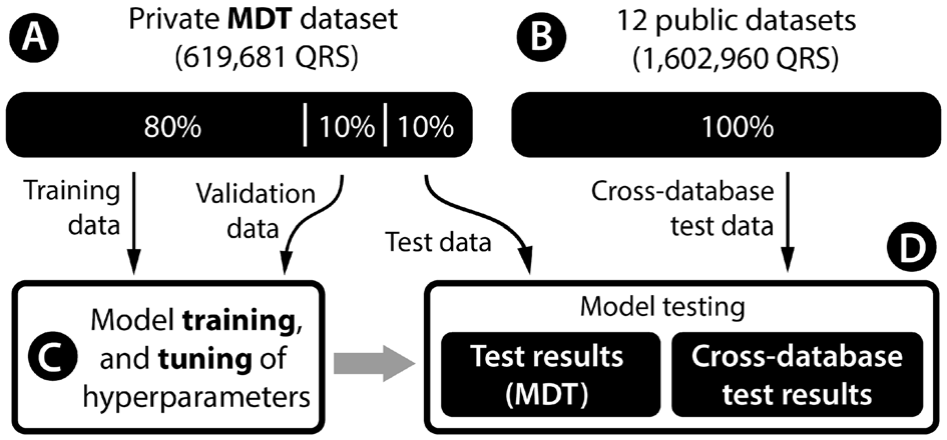
Dataflow in the presented study: private MDT data (**A**) were split into training, validation, and test subsets. Training and validation subsets were used to develop the proposed method (**C**). Next, the method performance was measured (**D**) using MDT private test data and data from twelve public databases (**B**). For the QRS classification task, only four databases were used.

We used private (Fig. 3A) and public (Fig. 3B) ECG datasets in this study. The anonymised, private ECG dataset was collected during routine ECG screening and, therefore, was not subject to the ethical committee by Czech law. This private dataset was used for the method development (Fig. 3C) and testing, and public datasets were used only for cross-database tests (Fig. 3D). The lead "I" was used if the dataset contained multiple leads. If it was not present, the first ECG lead was used.

### Private dataset

The private dataset MDT (Medical Data Transfer, s. r. o., Brno, Czechia) consisted of 12,111 single-lead Holter ECG recordings. Each recording was 45 s long, sampled at 200 Hz. Recordings were acquired from patients during usual daily activities and often contained a high amount of noise (Fig. 1B,D). We have developed a software tool, "QRS Marker". Two specialists with more than five years of experience with QRS detection and classification semi-automatically marked 619,681 QRS complexes in this tool. Next, data were split into training (80%), validation (10%), and testing (10%) datasets (Table 1) in an out-of-patient manner.

**Table 1:** QRS complex counts in the private MDT data for model training, validation and test.

Dataset	N	PAC	PVC	Total	Patients
Training	420,336	27,763	45,033	493,132	1,918
Validation	53,051	3,893	7,838	64,782	240
Test	52,795	4,910	4,062	61,767	240
Total	526,182	36,566	56,933	619,681	2,398

*N* normal sinus QRS, *PAC* premature atrial contraction, *PVC* premature ventricular contraction.

### Public datasets

We also used twelve public databases (1,602,960 QRS complexes from 3,050 recordings, sampling frequency from 128 to 1000 Hz) to test QRS detection performance (Table 2). Set of twelve public datasets contained both parts of public data from CinC/PhysioNet Challenge 2014^21^, CPSC-2019 database^22^, CYBHi database^23^ using later created annotations^11^, European ST-T database (EDB)^24^, St. Petersburg INCART database (available from PhysioNet^25^), Lobachevski University Database (LUDB)^26^, MIT-BIH arrhythmia database^12^, QT database^27^, MIT-BIH ST change database (STDB)^25^, MIT-BIH Supraventricular Arrhythmia Database^28^, and T-wave alternans dataset (TWADB) from CinC/PhysioNet/Challenge 2008^29^. These twelve databases (Table 2) were used for cross-database tests in the QRS detection task.

**Table 2:** Public data for cross-database tests.

Dataset	Recordings	QRS	Fs [Hz]
CinC 2014, part 1^21^	100	72,415	250
CinC2014, part 2^21^	100	75,711	250 + 360
CPSC-2019^22^	2,000	29,467	500
CYBHi^11,23^	126	18,841	1,000
EDB^24^*	90	790,565	250
INCART*^25^	75	175,907	257
LUDB^26^	200	1,829	500
MIT-BIH*^12^	48	109,494	360
QT^27^	105	86,995	250
STDB^25^	28	76,175	360
SVDB*^28^	78	146,769	128
TWADB^29^	100	18,792	500

*Data were used also for QRS classification.

Databases EDB, INCART, MIT-BIH, and SVDB contained QRS classes and were used for cross-database tests of QRS classification performance (Table 2, rows highlighted with *). The proposed method is designed to classify into normal beats, PAC and PVC; therefore, if the QRS complexes were classified in more detail (e.g., a paced beat), the closest possible option was selected (e.g., a normal beat).

### Training data augmentation

We randomly inverted each signal with a probability of 0.5 and cropped the signal to random 30 s, modifying the data for each batch. We used weighted oversampling to balance the counts of the QRS types we trained on.

## Method

**Figure 4:**
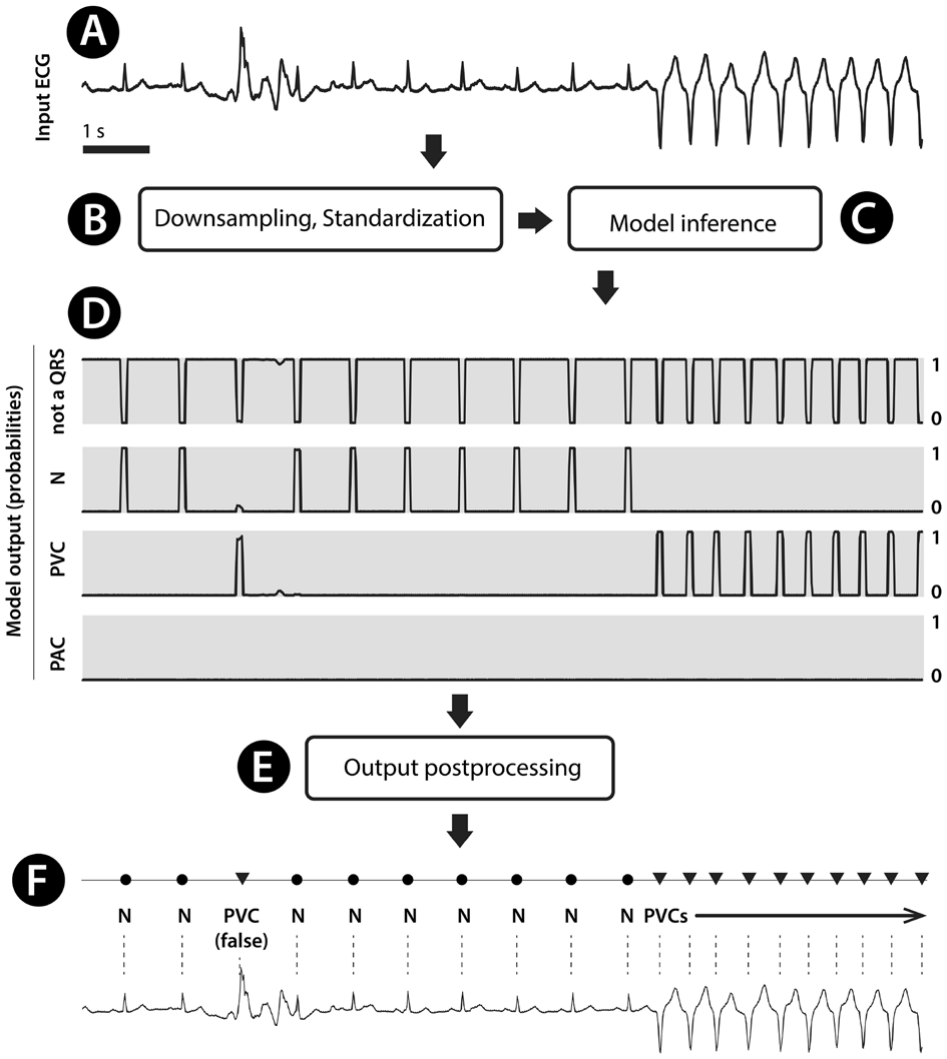
A workflow to detect and classify QRS with the proposed method. The ECG signal (**A**) is preprocessed (**B**) and imputted to the inference engine with the deep-learning model (**C**). The output of the model (**D**) consists of four probability vectors at the length of the input ECG signal. Output vectors are post-processed (**E**), and QRS annotation marks and classes are reported (**F**). N refers to normal sinus beat; PVC refers to premature ventricular contraction with one example of correctly detected but incorrectly classified QRS (the left-most PVC).

All experiments were performed in accordance with relevant guidelines and regulations. The method is designed to work as in Fig. 4. Raw ECG signal (Fig. 4A) is preprocessed (Fig. 4B), model inference (Fig. 4C) delivers probabilities of QRS classes (Fig. 4D), and post-processing (Fig. 4E) leads to resultant QRS with class information (Fig. 4F).

### Preprocessing

**Figure 5:**
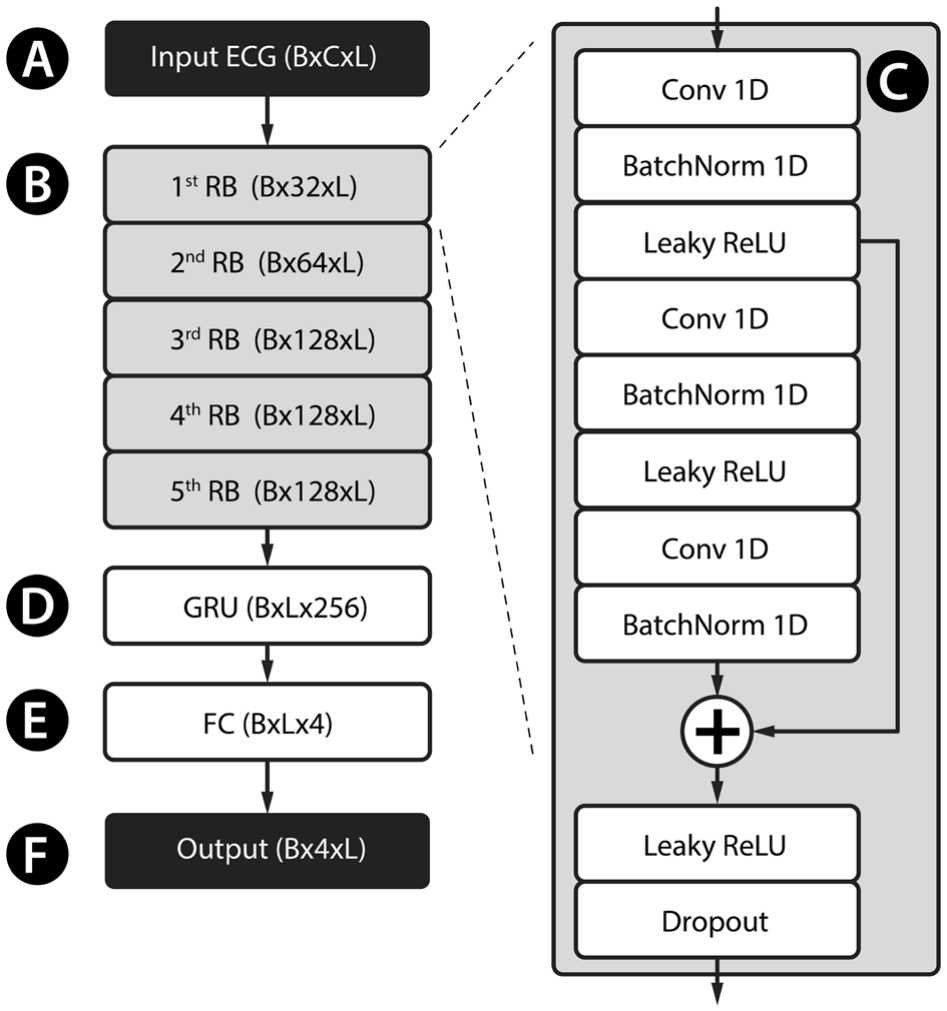
Architecture of the presented neural network. Standardized input ECG (**A**) enters the first Residual Block (RB). Five of RBs are chained together (**B**). RB is described in further detail in (**C**). Then the signal enters Gated Recurrent Unit (GRU, **D**) layer, Fully connected layer (FC, **F**) and transposition (**F**). B × C × L refers to batch size x channel count x signal length.

Before feeding the training signals into the model, we resampled the signal to 100 Hz and standardised the signal independently to have zero mean and unit variance (Fig. 5A). Target data (y) for the model were prepared as follows: each QRS location was widened to 10 samples to create a four-channel segmentation mask (as in Fig. 4D).

### Model architecture and training

The developed model consists of five residual blocks (Fig. 5B,C), a gated recurrent layer (Fig. 5D), and a fully connected layer (Fig. 5E). The model outputs a four-channel tensor (Fig. 5F). Each residual block consists of several convolutional layers. We used a batch size of 64, a cross-entropy loss function, an AdamW optimiser with a learning rate of 0.001, clipped the gradient L2 norm to 1.0, no weight decay.

### Post-processing

The network outputs the likelihood (Fig. 4F) of the four different QRS classes (no QRS, normal QRS, atrial QRS, ventricular QRS) for every input sample. We take the class with the maximum likelihood for every sample and post-process the resulting segmentation mask to get a list of the QRS peaks. First, we calculate the centers of the segmentation mask and save them into a list of potential peaks. Then, we remove lower peaks that are too close (< 0.15 s) to stronger peaks, as such a low distance between beats is physiologically improbable.

### Compared QRS detectors and used metrics

For comparison, we also evaluated used datasets by several publicly available QRS detection methods: by Elgendi^2^, Malik et al.^3^, XQRS detector from Python WFDB package^30^, and by Pan and Tompkins^1^. We also evaluated three-detectors from the python NeuroKit package—modified Engelse & Zeelenberg^6^, Hamilton detector^4^, and Kalidash detector^7^. Results for these detectors might differ from the performance reported by respective papers since we used all available data from all datasets; we implemented detectors by Elgendi^2^ and Malik^3^ using respective papers.

We used the F1 score to compare and evaluate results. A detected QRS complex was considered true positive when its annotation mark was closer than 0.1 s (inclusive) to an annotation mark prepared by an expert. The false positive or false negative cases were considered when a beat was missing in expert annotations or detected QRS complexes.

## Results

The model was built using the PyTorch framework^31^ and trained in 70 epochs using the private MDT dataset. We separately evaluated QRS detection performance and QRS classification performance; we also evaluated computational method performance.

### QRS detection performance

We received training, validation, and testing F1 scores of 0.991, 0.990, and 0.992 for the detection task using the MDT test set. We also provided a cross-database test to evaluate detection performance on twelve public datasets, showing a mean F1 score of 0.96 ± 0.06. The detection performance was compared to other methods using all twelve test datasets. We received a maximal mean F1 score of 0.961 using the proposed method, followed by the Malik method^3^ (0.955) and XQRS detector from the WFDB^30^ Python package. We also observed how the used databases were difficult for tested detectors. Overall F1 results by all detectors per database showed that the easiest database to detect was the STDB^25^ (0.979), the first part of PhysioNet/CinC challenge 2014^21^ (0.972) and the MITDB (0.958). On the other hand, the most challenging database for tested detectors was the second part of PhysioNet/CinC challenge 2014 (0.759), the SVDB (0.805), and the MDT test set (0.809).

### QRS classification performance

We evaluated the proposed method to classify QRS complexes into three groups—normal beat, premature ventricular contraction, and premature atrial contraction (Table 4—the last row). We reached an overall classification F1 performance of 0.96 and 0.74 for micro and macro computation in the MDT test set, respectively. Cross-database tests for QRS classification (Table 4, the first four rows) showed average micro and macro F1 scores of 0.95 ± 0.03 and 0.73 ± 0.06, respectively.

### Method computational performance

We measured the processing time of all compared methods using all testing datasets (excluding the CYBHi) to evaluate computational performance. The average processing time per record is shown in Fig. 7. The comparison was obtained using a computer with Intel® Xeon® Gold 6248R CPU running at 3.00 GHz. Data were supplied to algorithms one by one, and we disabled GPU, which disadvantaged the proposed method (Fig. 7).

## Discussion

**Figure 6:**
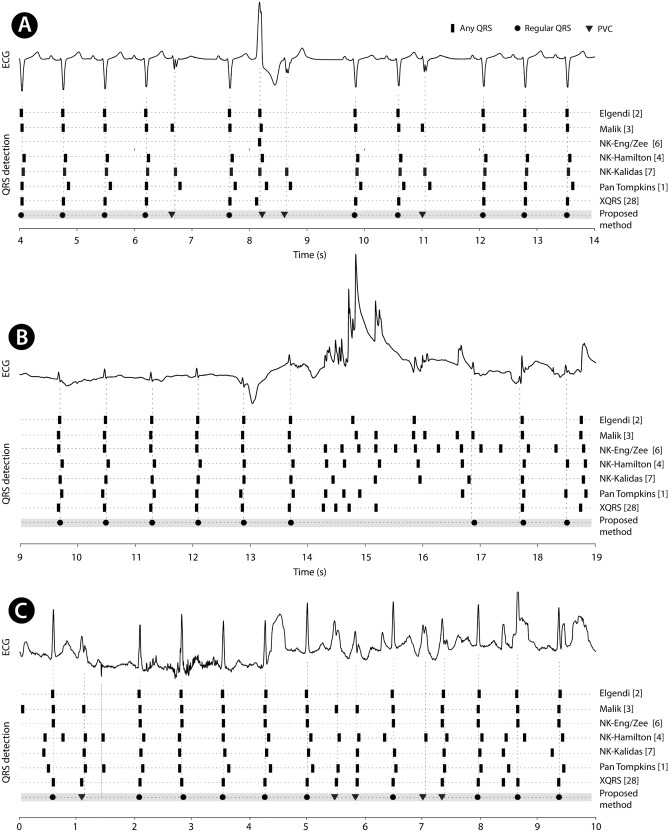
Non-trivial Holter ECG examples (parts of 30-s blocks) with QRS detection results by several methods. (**A**) Normal sinus rhythm with multifocal premature ventricular beats; (**B**) normal sinus rhythm, heavily disturbed; (**C**) normal sinus rhythm, disturbed, with premature ventricular beats. Bold black rectangles refer to regular QRS, filled triangles refer to premature ventricular contraction (PVC). Bold vertical at approx. second 1.3 refers to a questionable PVC combined with an artifact. Shown signals were not used for development nor testing of the presented method.

The presented method showed the highest overall QRS detection F1 score in compared methods (Table 3) when using all test datasets. We were generally focused on Holter ECG data acquired during usual daily activities, and we received the best score of tested methods in the MDT dataset. The highest overall score might reflect that we used a high amount of disrupted ECG data for training. Table 3 (the row "MDT") demonstrates how different methods can detect QRS in noisy data. Figure 6 shows examples of non-trivial Holter ECG and results of presented and compared detection methods. Figure 6A demonstrates that four methods overlooked PVCs with abnormally low amplitude; Fig. 6B shows how methods react to signal disturbance and how most of them capture noise instead of QRS if they are very close (19th second). Finally, Fig. 6-C demonstrates how non-usual PVC couplets and noise may confuse detectors.

**Table 3:** Resultant F1 scores for QRS detection using various detection methods and testing datasets.

Test dataset	Elgendi^2^	Malik^3^	EngZee (NK)^6^	Hamilton (NK)^4^	Kalidas (NK)^7^	Pan Tompkins^1^	XQRS^28^	The proposed method
CinC 2014 (Part 1)	0.998	0.998	0.969	0.830	0.994	0.993	0.998	0.999
CinC 2014 (Part 2)	0.798	0.815	0.682	0.722	0.717	0.707	0.798	0.827
CPSC-2019	0.900	0.911	0.668	0.768	0.845	0.782	0.900	0.944
CYBHi	0.965	0.962	0.523	0.911	0.446	0.937	0.949	0.972
EDB	0.974	0.987	0.867	0.524	0.970	0.428	0.974	0.994
INCARTDB	0.890	0.959	0.803	0.869	0.869	0.837	0.890	0.956
LUDB	0.893	0.871	0.845	0.675	0.886	0.777	0.893	0.887
MITDB	0.989	0.990	0.903	0.906	0.956	0.928	0.989	0.997
QTDB	0.973	0.987	0.933	0.799	0.979	0.631	0.973	0.997
STDB	0.973	0.995	0.907	0.980	0.990	0.995	0.973	0.997
SVDB	0.293	0.995	0.859	0.859	0.963	0.488	0.293	0.996
TWADB	0.953	0.976	0.850	0.863	0.945	0.814	0.953	0.970
MDT (private)	0.366	0.974	–	0.943	0.921	0.562	0.366	0.992
Overall mean ± std	0.843 ± 0.226	0.955 ± 0.054	0.817 ± 0.124	0.819 ± 0.119	0.883 ± 0.146	0.760 ± 0.180	0.943 ± 0.060	0.961 ± 0.057

*NK* refers to the Python package NeuroKit.

We also compared the presented method to the deep-learning method^11^ trained on the CYBHi dataset^23^ and tested on MIT-BIH^12^ dataset with an F1 score of 0.96. Our method slightly outperforms the compared method on MIT-BIH, but on the other hand, we used a significantly more complex network structure.

The important benefit of the presented method is that it classifies QRS complexes into three groups. Our results show that the weakest point of QRS classification is in the PAC class (Table 4). Further investigation revealed that in most cases, false PACs are generated inside blocks of atrial fibrillation where the presented method tends to report PACs. We also found incorrect classifications in long SVT runs (series of PACs running on high heart rate).

**Table 4:** Resultant test F1 scores for QRS classification.

Dataset	N	PAC	PVC	Total (Micro)	Total (Macro)
EDB	0.99	0.17	0.80	0.99	0.66
INCARTDB	0.95	0.65	0.69	0.92	0.76
MITDB	0.96	0.34	0.77	0.93	0.69
SVDB	0.98	0.62	0.79	0.95	0.81
MDT (test set)	0.97	0.92	0.94	0.96	0.74

**Figure 7:**
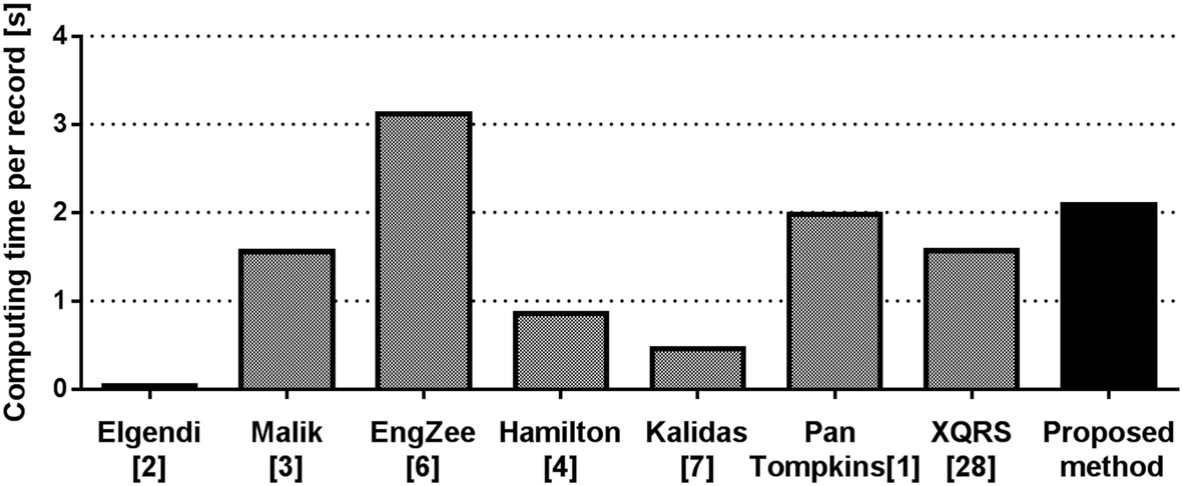
Comparison of average processing time per record (30-s long) in all test datasets (excluding the CYBHi dataset).

A limitation in comparison to most other methods is processing time, as shown in Fig. 7. However, this can be overpassed when the model uses a GPU during inference. In such a case, inference time can be decreased approximately 10–30 times depending on the specific hardware and batch size.

## Conclusion

We presented a novel deep learning method for QRS detection and classification in one inference step. The method was evaluated on twelve public datasets not used for model development. This cross-database test showed higher overall QRS detection performance than other compared methods. Furthermore, we showed how compared QRS detectors behave in non-trivial situations common in Holter ECG. We also demonstrated that both QRS classification and detection could be combined into one deep-learning model. Therefore, the usual processing chain to analyse heart rhythm can be simplified.

## Data Availability

Private data supporting this study's findings are available from Medical Data Transfer, s.r.o. (Brno, Czech Republic) but they are not publicly available. However, data are available from the authors upon reasonable request and with the permission of the Medical Data Transfer company.
